# Tumor-associated macrophages in bladder cancer: roles and targeted therapeutic strategies

**DOI:** 10.3389/fimmu.2024.1418131

**Published:** 2024-11-13

**Authors:** Yuanchun Ma, Ying Sun, Hongqian Guo, Rong Yang

**Affiliations:** ^1^ Department of Urology, Nanjing Drum Tower Hospital, Affiliated Hospital of Medical School, Nanjing University, Nanjing, China; ^2^ Department of Urology, Nanjing Drum Tower Hospital, Nanjing Drum Tower Hospital Center of Molecular Diagnostic and Therapy, State Key Laboratory of Pharmaceutical Biotechnology, Jiangsu Engineering Research Center for MicroRNA Biology and Biotechnology, Nanjing University Advanced Institute of Life Sciences (NAILS), Nanjing, China

**Keywords:** tumor-associated macrophage, tumor immune microenvironment, macrophage targeted therapy, bladder cancer, macrophage polarization

## Abstract

Bladder cancer (BC) is the ninth most common and “expensive” cancer in the world. Despite the availability of various treatment modalities such as chemotherapy, immunotherapy and surgery, the overall survival rate of patients with advanced bladder cancer remains low. As one of the most abundant infiltrating immune cells in bladder cancer, tumor-associated macrophages (TAMs) play an important role in the development of BC and in the standard regimen of intravesical BCG therapy. Targeting TAMs have achieved excellent results in clinical trials for a variety of other cancers, but few studies have been conducted for bladder cancer. Further exploration is still needed to develop TAM-related therapeutic strategies for BC treatment, which are expected to improve the therapeutic efficacy and life quality of patients. This review summarizes the relationship between TAMs in bladder cancer and disease staging, evolution, patient prognosis, and treatment outcome. Several potential TAM targets in BC are also pointed, which may help to inhibit tumor-promoting TAMs and provide new therapeutic approaches for advanced BC.

## 1 Introduction

Bladder cancer is the ninth most common cancer in the world and one of the most common genitourinary malignancies. Urothelial carcinoma is the most common type of bladder cancer, of which approximately 75% of patients present with non-muscle invasive bladder cancer (NMIBC), 20% with muscle invasive bladder cancer (MIBC), and the other 5% with metastatic disease (1). Despite the high cost of treatment, the clinical prognosis of bladder cancer remains poor. Transurethral resection of bladder tumor (TURBT) is the primary treatment for low-risk NMIBC, and TURBT combined with BCG intravesical instillation is the gold standard treatment for medium- and high-risk NMIBC, however, 50-70% of NMIBC recur within 5 years despite treatment, of which 10% to 30% develop MIBC or metastatic diseases (2). Compared to NMIBC, the progression and metastasis of MIBC is more rapid, and the overall prognosis is poorer. The standard treatment for MIBC is radical cystectomy combined with lymphadenectomy, which is usually preceded by neoadjuvant chemotherapy, yet the 5-year survival rate is only about 60% after operation (3).

**Figure 1:**
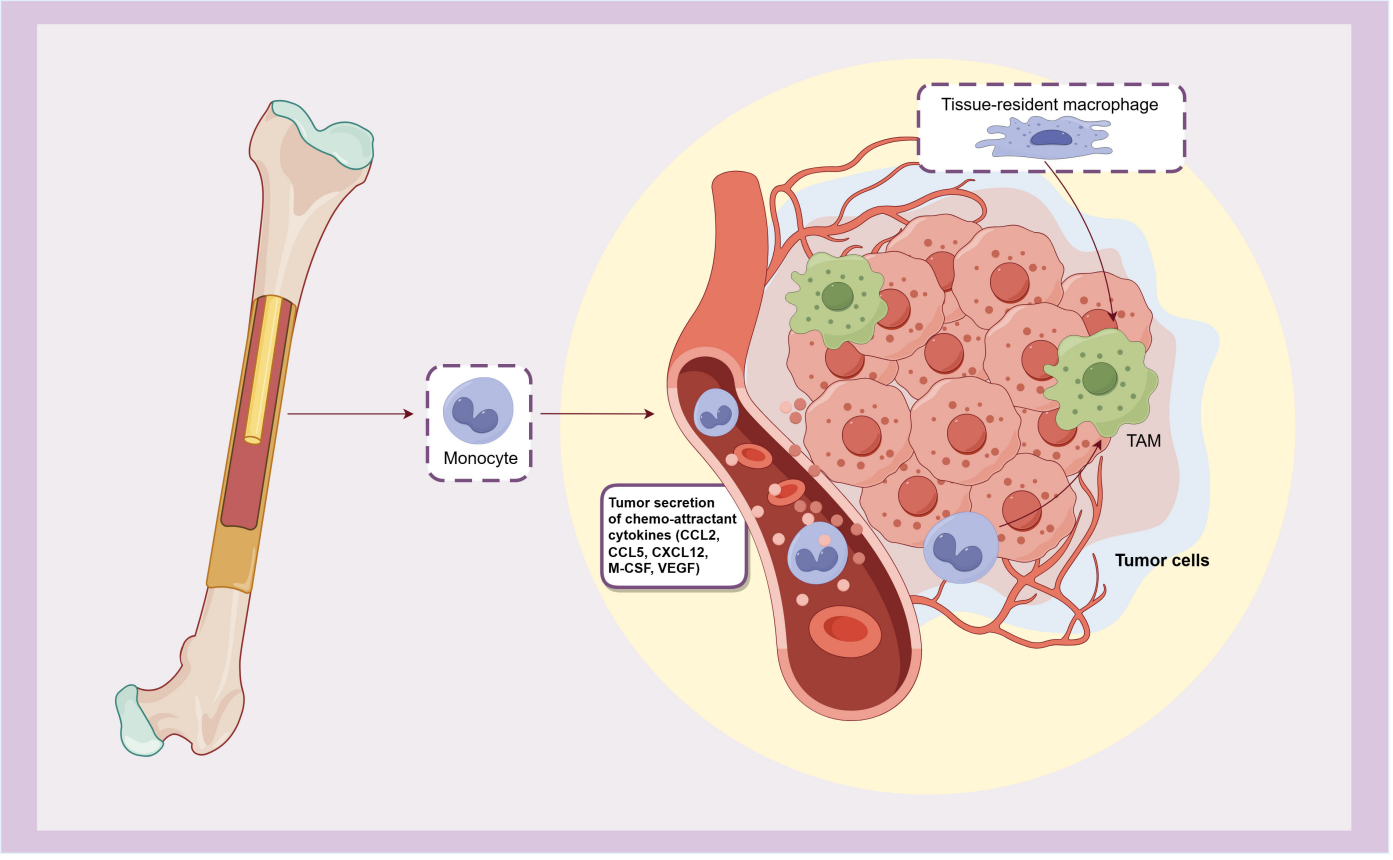
The origin of TAMs.

The tumor microenvironment (TME) of bladder cancer plays an important role in its growth, invasion and metastasis. By regulating the tumor immune microenvironment, immune checkpoint inhibitors (ICI) such as BCG, anti-PD-1 and PD-L1 antibodies are effective in the treatment of bladder cancer. As the most infiltrating inflammatory cells in bladder cancer TME, macrophages play an extremely important role in its occurrence and development. Following the release from the bone marrow, monocytes enter the circulation and are subsequently recruited in the TME by various chemokines (such as M-CSF, CCL2, CCL5, CXCL12, and VEGF) secreted by tumor cells, and then differentiate into tumor associated macrophages (TAMs). Additionally, tissue-resident macrophages can also undergo differentiation into TAMs when stimulated by tumor-related and other TME-injury factors (4, 5) (**Figure 1**). TAMs exhibit notable plasticity and heterogeneity, playing a pivotal role in the tissue homeostasis of TME. They contribute to angiogenesis and produce various immunomodulatory factors, affecting the initiation, progression, treatment response, and metastasis of tumor. Ultimately, these dynamics frequently culminate in tumor deterioration and unfavorable prognosis.

TAMs originate from monocytes that released from bone marrow and recruited by chemokines secreted by tumors, or differentiate from tissue-resident macrophages.

## 2 Phenotype and polarization of macrophages

In the case of infection and tissue damage, myeloid derived monocytes are recruited to target sites and differentiated into macrophages and dendritic cells. Macrophages play an important role in the innate and adaptive immune system by phagocytizing pathogens and apoptotic cells, presenting antibodies, and secreting various inflammatory factors and cytokines.

Macrophages have strong plasticity and are activated by receiving microenvironmental signals, and mainly differentiate into two distinct phenotypes: classical activated macrophages (M1 phenotype), and alternatively activated macrophages (M2 phenotype). Among them, M1 macrophages mainly participate in the immune response of type I helper T cells (Th1), which is responsible for inducing inflammation and killing bacteria and tumors. M2 macrophages are mainly involved in the Th2 type immune response, which can inhibit inflammation, promote wound healing and tumor progression, leading to the development of a variety of tumors and poor prognosis, so it is considered to be pro-tumor macrophage (6).

### 2.1 M1 macrophages

**Figure 2:**
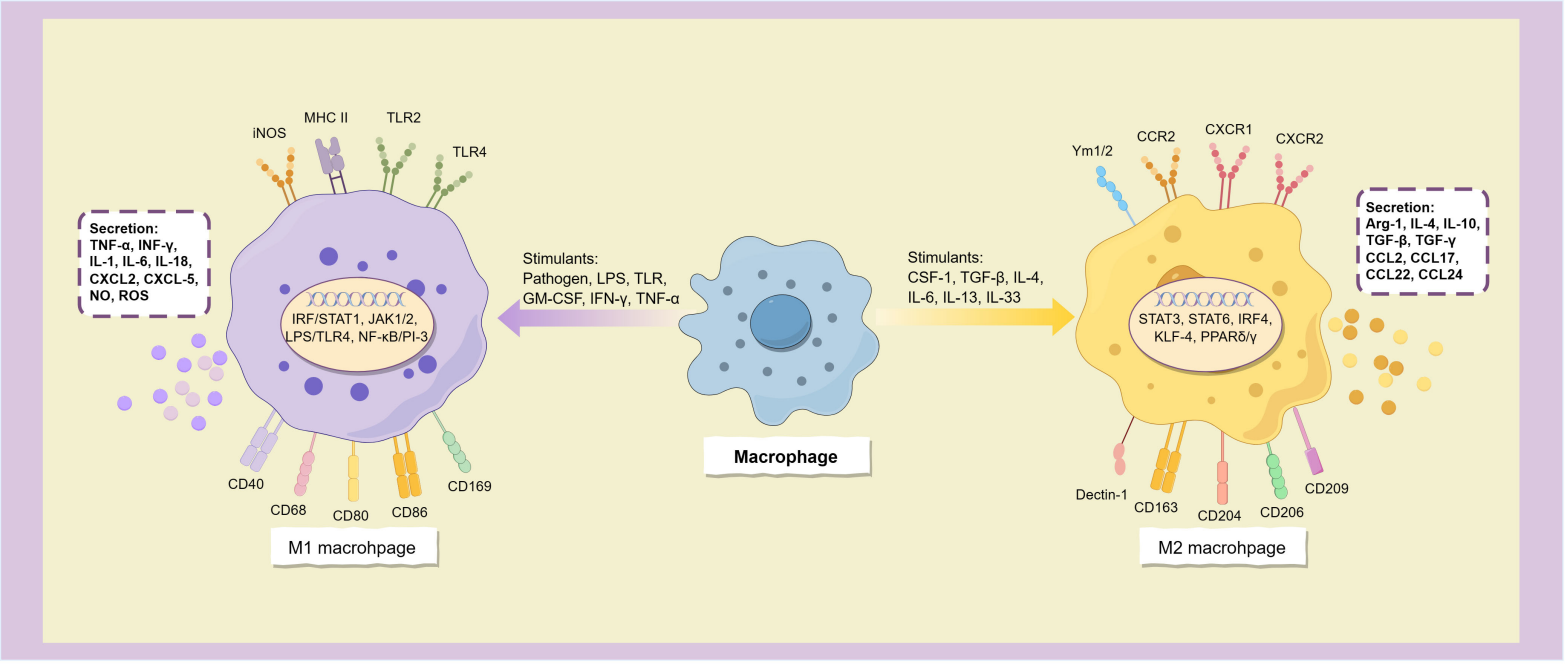
Macrophages polarize into two subtypes.

Classical activated macrophages (M1 macrophages) are typically stimulated by infection or tissue injury and are characterized by a surface phenotype of high levels of MHC II, TLR-2, TLR-4, iNOS, and co-stimulatory molecules including CD40, CD68, CD80, CD86, and CD169 (7, 8). When stimulated by a variety of molecules such as pathogens, lipopolysaccharides (LPS), Toll-like receptors (TLR), granulocyte macrophage colony-stimulating factor (GM-CSF), and Th1 cytokines (such as IFN-γ and TNF-α), macrophages polarize to the M1 phenotype. This process depends on a variety of signal transduction pathways such as IRF/STAT1, JAK1/2, LPS/TLR4, and NF-κB/PI-3 kinase pathway (9), among which NF-κB is a key transcription factor for M1 polarization, and many other transcription factors, such as STAT1, STAT5, IRF3 and IRF5, are also involved in regulating the expression of M1 gene in macrophages (7) (**Figure 2**).

M1 macrophages have strong antigen-presenting activity, and can release a variety of cytokines and chemokines, including TNF-α, INF-γ, IL-1, IL-6, IL-12, IL-16, IL-18, IL-23, and Th1 cell chemokines CXCL1-3, CXCL-5, and CXCL8-12 (10), and also secretes a large amount of NO and reactive oxygen species (ROS) (11). These inflammatory factors not only have the ability to activate immune cells such as Th1, Th17 cells and NK cells, but also induce more macrophages to polarize toward the M1 subtype (8), which allows them to initiate inflammatory responses and enhance tumor killing effects.

### 2.2 M2 macrophages

M2 macrophages can be activated by parasitic or fungal infection, immune complexes, apoptotic cells, and multiple immune factors such as macrophage colony-stimulating factor (M-CSF or CSF-1), TGF-β, IL-4, IL-13, IL-33, and IL-25 (10). It is characterized by high expression of CD163, CD204, CD206 (mannose receptor), CD209 (DC-SIGN), Dectin-1, FIZZ1, Ym1/2, CCR2 and CXCR1-2 (11, 12), and low expression of MHC II (13).

Alternatively activated macrophages have the opposite cytokine expression profile to the classical activated phenotype, secreting low levels of pro-inflammatory cytokines (such as IL-1, IL-6, IL-12, IL-23, and TNF-α) and releasing more anti-inflammatory and immunosuppressive mediators, such as arginase-1 (Arg-1), IL-4, IL-10, IL-1RA, TGF-β and TGF-γ (7, 12, 13), and express many chemokines such as CCL1-2, CCL17-18, CCL22 and CCL24 (7), thereby inhibiting immune response, relieving inflammation, repairing damaged tissues, promoting angiogenesis, and providing conditions for immune escape of tumors (**Figure 2**). The surface markers of M2 macrophages are similar to the dominant population of macrophages in the tumor microenvironment, and thus are often considered to be the main components of TAM.

Macrophages mainly polarize into M1 and M2 phenotypes when activated by stimulus signals, these two distinct subtypes express different surface molecules and cytokines, participating in distinct immune responses.

Based on variations in cell surface markers, secreted cytokines, and biological functions, the known M2 macrophages can be categorized into four subclasses: M2a, M2b, M2c, and M2d. Specifically, M2a macrophages are activated by IL-4 or IL-13 and primarily participate in the anti-parasitic Th2 response (6). M2b macrophages, on the other hand, are activated by immune complexes, LPS, IL-1β, and IL-1R ligands, and contribute to immune response and inflammation regulation by releasing pro-inflammatory and anti-inflammatory cytokines such as TNF-α, IL-1β, IL-6 and IL-10 (7). The surface of M2c macrophages is characterized by the presence of CD163, CD206, and RAGE receptors, which are activated by glucocorticoids, TGF-β, and IL-10. This subtype of macrophages secretes the anti-inflammatory factor IL-10, the pro-fibrotic factor TGF-β, as well as CCL16, CCL18, and mer receptor tyrosine kinase (MerTK). Additionally, M2c macrophages play a role in angiogenesis, tissue repair, and the phagocytosis of apoptotic cells (6, 14). M2d macrophages are primarily activated by TLR antagonists, adenosine, and IL-6, and they secrete vascular endothelial growth factor-A (VEGF-A), TGF-β, IL-10, and IL-12. These secreted factors contribute to the acceleration of angiogenesis and tumor progression (7, 14).

IL-10 and IL-4/IL-13 induce macrophages to polarization towards M2 phenotype through activation of STAT3 and STAT6, respectively. This process is regulated by transcription factors IRF4, KLF-4, PPARδ and PPARγ (7, 11). Additionally, toll-like receptors and immunoglobulins can stimulate the alternative activation of macrophages. Furthermore, tumor-produced factors including IL-6, TGF-β and CSF-1 can also induce the polarization of TAM towards the M2 phenotype (8, 15). In addition, epigenetic modification also plays a crucial role in the differentiation and activation of macrophages. Histone deacetylase (HDAC) serves as epigenetic modifier that regulate the differentiation of M2 macrophages (16). Methylation of histones H3K4, H3K27 and H4R3 also exert a significant influence on the polarization and activity of macrophages (16). Notably, the H3K27 demethylase KDM6A is frequently found mutated in bladder cancer, and its deficiency upregulates cytokines and chemokines in mouse tumor models, promoting M2 polarization of macrophages, and collaborates with p53 dysfunction to contribute to bladder cancer (17).

## 3 TAMs promote the progression and metastasis of bladder cancer

Distinguishing itself from the well-defined M1 and M2 macrophage subtypes, TAMs display a dynamic phenotype spectrum that responds to local microenvironmental stimulation. During the early stage of tumorigenesis, TAMs exhibit functionalities akin to M1 macrophages, produce IFN, ROS and a variety of pro-inflammatory cytokines, effectively activating cytotoxic T cells to induce anti-tumor immune response. However, as the cancer progression advances, TAMs undergo a gradual phenotypic transformation, transitioning toward an M2-like phenotype through programming and reprogramming, resulting in the establishment of an immunosuppressive TME and tumor immune escape (8, 18).

In macrophages co-cultured with bladder cancer cells, factors associated with the M2 subtype, such as CD163, CCL2, IL-10 (12), and DC-SIGN (19), exhibited significantly elevated expression levels. This observation suggests that the bladder cancer-associated TAMs predominantly assume an M2-like polarization phenotype, thereby exerting tumor promoting effects akin to M2-type macrophages. The correlation between TAM and the grade and stage of bladder cancer has been consistently validated through a large number of TCGA cohort studies (2, 20–24). TAM infiltration, particularly that of M2-like TAMs, has been strongly associated with an unfavorable prognosis and disease progression in bladder cancer, including diminished progression-free survival (PFS) and overall survival (OS), and can serve as a valuable prognostic indicator of recurrence in patients with NMIBC (25–27).

In comparison to NMIBC, MIBC is associate with greater inherent danger and a poor prognosis, which may be attributed, in part, to differences in the infiltration pattern of TAM between the two. In NMIBC, TAMs primarily localize at the interstitial margin of the tumor, whereas in MIBC, TAMs predominantly infiltrate the tumor areas (6). In addition, there is a higher overall macrophage infiltration observed in MIBC compared to NMIBC, with squamous epithelial carcinoma, a predominant subtype of MIBC, exhibiting a greater infiltration of M2-like TAMs (19, 26), which may help MIBC to become more aggressive and malignant.

**Figure 3:**
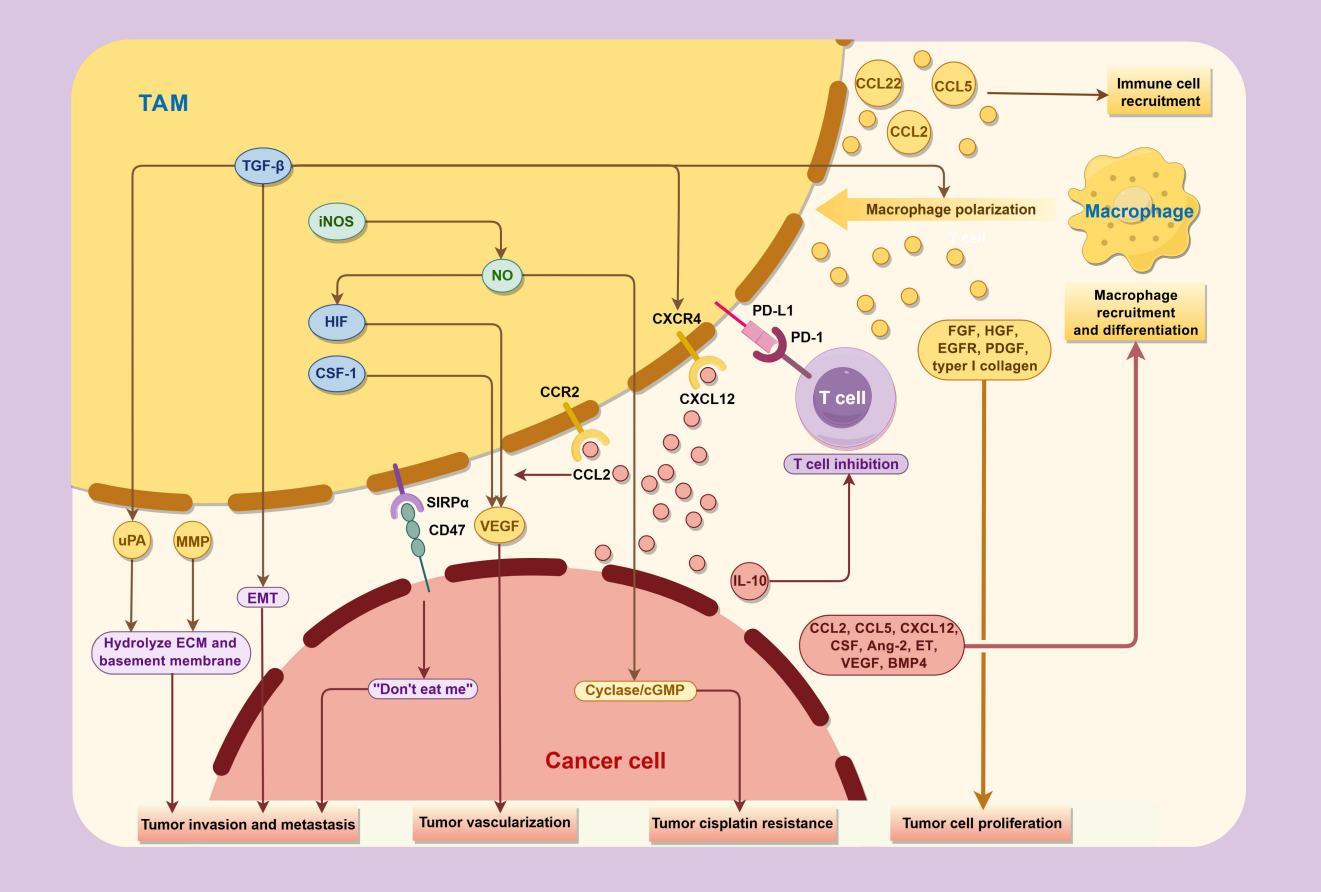
Interactions between TAMs and cancer cells in the tumor microenvironment.

The significant correlation observed between TAMs and tumor prognosis underscores the pivotal role of TAMs in tumor development. Previous studies have elucidated that TAMs can promote tumor initiation, progression, and metastasis though multifaceted mechanisms, including the production of tumor growth factors, facilitation of peritumoral angiogenesis, release of proteases and other molecules capable of remodeling the extracellular matrix, and secretion of immunosuppressive mediators, all of which collectively impair the host immune system’s antitumor capabilities (**Figure 3**).

TAMs promote the progression and metastasis of bladder cancer through various cytokines, while concurrently being regulated by factor secreted and expressed by tumor in TME.

### 3.1 TAMs express a spectrum of tumor promoting factors

The M2-like TAMs exhibit reduced secretion of pro-inflammatory factors such as IL-6 and TNF-α, while concurrently upregulating various anti-inflammatory factors including IL-10, TGF-β, and PGE2. This profile maintains the immunosuppressive milieu within the tumor microenvironment. Simultaneously, TAMs secret a variety of chemokines such as CCL2, CCL5 and CCL22, recruiting Th2 cells, Treg cells, bone marrow-derived suppressor cells (MDSC), and macrophages themselves to the tumor site, which amplifies the immunosuppressive Th2 immune response and promotes the differentiation of additional macrophages into the immunosuppressive TAM phenotype. Excessive Th2 cells and M2-type macrophages recruit fibroblasts, leading to the overproduction of extracellular matrix (ECM) deposition through IL-4, IL-13 and fibroblast growth factor (FGF) (19). This process induces pathological fibrosis in TME, and play a crucial role in the progression and recurrence of bladder cancer.

In addition, TAMs also produce a variety of factors that directly stimulate tumor cell proliferation and motility, including FGF, hepatocyte growth factor (HGF), epidermal growth factor receptor (EGFR) family ligands, platelet-derived growth factor (PDGF) and TGF-β (28). TAMs can also promote the sustained proliferation of bladder cancer cells through secreting type I collagen, which can activate the integrin α2β1/PI3K/AKT signaling pathway. Traditional chemotherapy drugs combined with integrin α2β1 inhibitors have shown powerful anti-cancer effects (29).

### 3.2 TAMs promote tumor angiogenesis

TAMs produce various pro-angiogenic molecules that exert an important role in the development of bladder cancer, including vascular endothelial growth factor (VEGF), epidermal growth factor (EGF), thymidine phosphorylase (TP), TNF-α, TGF-β, IL-1, IL-8, NO, and chemokines such as CCL2 and CXCL8 (30).

Overexpression of VEGF has been detected in most cancers, including bladder cancer. It is primarily produced by tumor cells, macrophages, and T cells within the tumor tissue (31), and can in turn skew the differentiation of monocytes towards CD163^high^CD86^low^IL-10^high^ M2-like macrophages (32), which is associated with tumor grade and prognosis. The upregulation of VEGF expression by TAM is mediated by CSF-1 and hypoxia-inducing factor (HIF) (28), and is further enhanced in the hypoxic tumor microenvironment, where VEGF attracts monocytes through VEGF receptor (flt-1) (33), thus creating a positive feedback loop of tumor vascularization. VEGF can also stimulate EMT by activating the transcription factor nuclear factor (erythroid-derived 2)-like 2 (Nrf2) in an ERK1/2-dependent manner (34), and the hypoxia induced by Nrf2 activation can in turn regulate HIF-1α/VEGF signaling pathway and modulate angiogenesis (35). In a clinical report on invasive bladder cancer, researchers found that the location of endothelial Per-ARNT-Sim (PAS) domain protein-1 (EPAS-1)/hypoxia-inducible factor-2α (HIF-2α) expression was identified mainly in TAMs, which is positively correlated with the expression of VEGF (36).

Among the VEGF family members, angiogenic VEGF−A and lymphangiogenic VEGF−C (37) were found to directly correlate with the M2-TAMs markers CD68 and CD163 (38), contributing to the progression of tumors. The use of bevacizumab (VEGF antibody) (39) and ramucirumab (VEGFR2 antibody) (40), in combination with chemotherapy, has exhibited promising results in Phase II clinical trials for locally advanced or metastatic bladder cancer.

Throughout the development of bladder cancer, TAMs assume a crucial role in carcinogenesis associated with inflammation by releasing large amounts of NO via inducing nitric oxide synthase (iNOS) (41). NO can subsequently upregulate the transcription factor HIF, thereby promote the expression of VEGF and other pro-angiogenic molecules, and promote tumor growth and infiltration by promoting vasodilatation (18). Nevertheless, it is worth noting that at high concentration, NO exhibits pro-apoptotic function, resulting in tumor suppression (42). M1-type macrophages in the TME generate significant amounts of NO, promoting tumor cell apoptosis and M1-like polarization of TAMs (18). In contrast, M2-type TAMs exhibit reduced iNOS and NO production, leading to the tumor resistance to cisplatin-induced apoptosis via the activation of the guanylate cyclase/cGMP system and the reduce of acid sphingomyelinase transporter Synt4 (43).

### 3.3 TAMs promote tumor invasion and metastasis

TAMs also play a crucial role in tumor invasion and metastasis. TAMs in bladder cancer secrete multiple matrix metalloproteinases (MMPs), including MMP-2, MMP-7, MMP-9, and MMP-12). When activated, these proteinases can hydrolyze various components of the basement membrane and ECM, such as type IV collagen, thus promoting the migration and invasion of cancer cells (33, 44).

Urokinase-type plasminogen activator (uPA) exhibits a similar effect, being overexpressed on TAMs and activated by CSF-1 receptor signaling and TGF-β. Upon binding to its receptor uPAR, uPA activates plasminogen to plasmin, leading to the hydrolysis of the basement membrane and ECM, thereby impacting angiogenesis and cell migration, ultimately promoting the metastasis of bladder tumor (28, 45). Chen et al. intravesically injected a high concentration of plasminogen activator inhibitor-1 (PAI-1) into the bladder of a rat bladder model, resulting in a 53% reduction in tumor size and a decrease in muscular invasion, which suggesting the potential of uPA as a therapeutic target for bladder cancer (46).

There may also be fusion between macrophages and tumor cells in bladder cancer TME, contributing to increased tumor cell heterogeneity and promoting metastasis. Carolina Rubio et al. co-cultured bladder cancer cell lines with human macrophages and detected fused myeloid-tumoral hybrid cells. Compared with unfused cancer cells, these fused cells underwent phenotypic changes and thus acquired myeloid cell characteristics, providing a selective advantage for tumor clones with enhanced adaptability and metastasis potential, consequently resulting in increased cell migration behavior (47–49).

Another pivotal pathway driving metastasis involves epithelial-mesenchymal transformation (EMT), a process encompasses the influence of multiple cytokines, with one of the principal drivers being TGF-β. TGF-β originates from TAMs or the tumor itself, which not only triggers EMT but also promotes differentiation of inactivated macrophages into an immunosuppressive phenotype (47, 50). Additionally, a variety of other cytokines secreted by M2-TAM can also promote EMT, including ZEB1, SNAL1, VIM and TWIST1. Meanwhile, the expression of EMT-associated long non-coding RNA (lncRNA) within the matrix also affects the infiltration of T cells and M2-TAM (51). Through these pathways, bladder cancer cells acquire a mesenchymal phenotype, thereby enhancing tumor mobility and metastasis.

### 3.4 TAMs inhibit anti-tumor immune response

Macrophages play a pivotal role in regulating the body’s immune response by secreting a series of factors. M1 macrophages promote the polarization and recruitment of Th1 cells by expressing cytokines and chemokines such as IL-12, CXCL9 and CXCL10. Conversely, M2 macrophages express chemokines like CCL17, CCL22 and CCL24, which recruit Treg cells and Th2 cells that inhibit inflammation and amplify Th2 immune response. Research conducted in breast cancer has suggested the significance of TAMs in shaping T cell phenotypes in TME, and the long-term interaction with TAMs reducing the motility of CD8+ T cells and the ability to invade cancer nests (52). Similar phenomenon has been found in bladder cancer, where the high expression of PD-L1 on circulating monocytes and TAMs in bladder cancer patients promotes T cell apoptosis and inhibits T cell proliferation, and this expression is further upregulated by IL-10 secreted by bladder cancer cells through STAT3 signaling pathway, ultimately establishing a cycle in TME leading to the reduction in cytotoxic T cells (53).

TAMs also promote immune evasion of tumors through the SIRPα-CD47 signaling axis. The transmembrane protein CD47 is overexpressed in bladder cancer cells but is absent in normal urothelium. Furthermore, TAMs expressing the signal regulatory protein α (SIRPα) are enriched in MIBC specimens (54). These SIRPα+ TAMs recognize CD47 on tumor cells, thereby activating the “don’t eat me” signaling pathway and counterbalance the pro-phagocytic signal calreticulin (55), leading to the inhibition of macrophage-mediated phagocytosis of tumors and is strongly associated with immune escape through T cell depletion (56, 57).

In addition, CD276, a molecule highly expressed in bladder cancer TAMs, also plays a role in suppressing tumor immunity. This is achieved by activating the lysosomal signaling pathway and the transcription factor JUN, thereby regulating the expression of AXL and MerTK and eventually leading to enhanced efferocytosis in TAMs. Knocking out CD276 of TAMs blocks this efferocytosis and enhances the expression of the major histocompatibility complex class II (MHCII) in TAMs, thereby inhibiting the immune escape of tumors (58).

## 4 TAMs are regulated by tumor microenvironment

Macrophage recruitment and differentiation is orchestrated by various factors in TME, including monocyte chemoattractant protein-1 (MCP-1 or CCL2), CCL-5, CXCL12, colony-stimulating factor (CSF), angiopoietin-2 (Ang-2), endothelin (ET), and VEGF (59).

Chemokines CCL2 and CCL5 are produced in various cancers and server as pivotal factors in the recruitment of mononuclear/macrophage cells to the tumor microenvironment, especially in tumor metastasis (59, 60). For example, the expression of CCL2 is regulated by lncRNA LNMAT1 in bladder cancer (37), which recruits TAM and induces the polarization of M2-TAMs via the CCL2-CCR2 axis, while also stimulates its autocrine circulation, and up-regulates the secretion of VEGF-C thereby fostering lymphogenesis and lymphatic metastasis, which is significantly correlated with the grade and stage of bladder cancer (61). Therapeutic agents targeting CCL2 have demonstrated therapeutic effects in preclinical cancer models (62).

Several chemokines within the CXCL family, such as CXCL1, CXCL2, CXCL5, CXCL8 (IL-8), and CXCL12, also play a crucial role in the recruitment of myeloid cells and TAM in bladder cancer, and are associated with tumor grading, staging, and metastasis (2). *In vitro* chemotaxis experiments demonstrated that bladder cancer cell line J82 induced MDSC migration via the CXCL2/IF-CXCR2 signaling pathway (63). CXCL12 and CXCR4 are not expressed in normal bladder tissue, but exhibit highly expression in bladder carcinomas (64). In addition to chemotaxis, CXCL12 and CXCR4 can induce monocyte differentiation into TAMs, which significantly increases tumor invasiveness (65). TGF-β, secreted by tumors and macrophages, upregulates CXCR4 expression on the surface of TAMs. These TAMs are attracted into blood vessels by CXCL12+ fibroblasts surrounding the blood vessels and differentiate into perivascular macrophages, which facilitate tumor metastasis and assist the migration of cancer cells into the bloodstream (66).

Macrophage colony-stimulating factor (M-CSF or CSF-1), produced by various cells including tumor cells, macrophages and fibroblasts, is a potent chemotactic factor of mononuclear macrophages and drives the differentiation of macrophages into immunosuppressive M2 phenotype within the TME (67), associated with unfavorable clinical prognostic indicators (28). Similarly, the upregulation of G-CSF is linked to tumor cell proliferation, angiogenesis, M2 macrophage recruitment, and enhanced EMT in bladder cancer. Conversely, GM-CSF enhances macrophage antigen presentation and immune responsiveness, and is associated with the inhibition of lymphogenesis and M2 macrophage recruitment (68). Mechanistically, GM-CSF can re-educate macrophages to secrete VEGF receptor-1 (sVEGFR-1), which binds and inactivates VEGF and blocks angiogenesis. In addition, administration of GM-CSF can also cause the transformation from M2 to M1 polarization in TAMs, thus affecting TAMs behavior and tumor metastasis (69).

Tumor progression leads to a hypoxic state within TME, further enhancing the recruitment of macrophages through upregulation of HIF-1, HIF-2, Ang-2, ET and VEGF. This hypoxic microenvironment also triggers macrophages to increase their secretion of pro-angiogenic growth factors and chemokines, such as VEGF, FGF2, TNF-α, MMP7 and MMP9, and stimulates them to transform into angiogenic M2 phenotype (59, 70). Additionally, tumor cells in hypoxic environment also release tumor suppressor protein M (oncostatin M) and eosinophilic chemotactic factor (eotaxin or CCL11), which promote macrophage infiltration into TME and polarization into M2-like phenotype driven by HIF-1α (11).

After being recruited to anoxic TME, TAMs encounter impediment in migration due to macrophage migration inhibitor factor (MIF), and is therefore trapped in the anoxic region of the tumor (71). MIF is widely expressed in various cell types, including macrophages, tumor cells, bladder epithelial cells, and others. It acts as a tumor promoting factor for bladder cancer, prostate cancer and other urinary system malignant tumors, mainly via the transmembrane receptor CD74. It facilitates the expression of multiple pro-tumor cytokines (including MCP-1, CXCR4, IL-6, and IL-8), mediates tumor angiogenesis, and may further promote cancer cell proliferation by stimulating the release of PGE2, thus contributing to the development of bladder cancer and chemotherapy resistance (71). Inhibition of MIF with hyaluronic acid, anti-MIF antibodies, or MIF inhibitors has demonstrated reduced bladder cancer cell proliferation and cytokine expression *in vitro*, while MIF inhibitor-treated MIBC mice also resulted in lower tumor burden and microvascular densibility (72). In addition, MIF has also been implicated in the M2-to-M1 polarization shift of macrophages. Small molecule MIF antagonist 4-IPP (73) and CD74 antagonist C36L1 (74) have both been found to induce the repolarization of M2-like macrophages in mouse melanoma towards M1-like phenotype, and meantime stimulate the immune activity of DC cells and T cells.

Macrophages recruited to TME are induced to undergo polarization towards immunosuppressive M2 phenotypes by a variety of signaling molecules produced by cancer cells, including lactic acid produced by tumor metabolism, multiple miRNAs and lncRNAs, CSF-1, CCL2, CCL3, and CCL14, and bone morphogenetic protein 4 (BMP4) (8).

Tumor cells metabolize glucose primarily through glycolysis, resulting in increased secretion of lactic acid as a by-product (Warburg effect). *In vitro* experiments have demonstrated that bladder cancer cells reprogram macrophages to M2 phenotype (75) in a manner depending on the flow of cancer cells-lactate, a process mediated by HIF-1α or nucleoid factor 2 (Nrf2). Furthermore, lactate has been shown to induce macrophage migration, consequently augmenting the density of TAMs in the tumor tissue (34, 76, 77).

As a driver of malignant cell evolution, lncRNA is also involved in regulating the polarization of macrophages in TME. lncRNA CCAT1 has been found to be an important regulator of M2 polarization and tumor cell invasion, overexpressed in multiple cancer types, which enhances tumor cell proliferation, invasiveness, and migration, and is associated with poor prognostic outcomes (78). Conversely, some lncRNAs exhibit anti-tumor activity. For instance, LINC00702 can inhibit the secretion of inflammatory factors by M2-TAMs and suppress tumor cell proliferation through the upregulation of DUSP1. Interestingly, LINC00702 has low expression in bladder cancer tissue (79).

The regulatory effect of miRNA on TAMs is usually accomplished by exosomes produced by tumor cells. Following uptake by macrophages, exosome miR-21 secreted by bladder cancer T24 cells and exosome miRNA secreted by MB49 cells facilitate the polarization of these microphages towards M2 phenotype. This effect is achieved through the downregulation of PTEN and PI3K/AKT/STAT3/6 signaling pathways, thereby promoting the growth of bladder tumors in murine models (50, 80). Among them, miR-21, functioning as a downstream molecular switch governing macrophages activation, is upregulated by CSF-1R pTyr-721 signal, thus mediates the inhibition of M1 phenotype and enhancement of M2 phenotype gene expression (81).

BMP4, secreted by bladder cancer cells, also plays a conducive role in fostering the differentiation of mononuclear-macrophages into M2-like phenotypes, which is closely associated with EMT and tumor invasion. In addition, BMP4 promotes the differentiation of urothelial cells and, interestingly, inhibits proliferation while promoting differentiation in tumor cells. To counteract this impact of BMP4, bladder cancer cells downregulate the BMP type II receptor (BMPR2) on their surface through the secretion of miR-21, thereby rendering the tumor resistant to the inhibitory effects of surrounding BMP (82).

## 5 TAMs and bladder cancer treatment

Highly invasive TAMs not only contribute to tumor progression and metastasis, and establish an immunosuppressive microenvironment, but also closely linked to tumor resistance against anticancer drugs and recurrence risk. Research have revealed a significant association between a high infiltration of M2-TAMs and non-responsiveness to chemotherapy (2). The polarization status of TAMs and Th1/Th2 balance in TME prior to treatment may also affect the clinical response to BCG therapy and recurrence risk. Patients with higher degrees of TAM infiltration before treatment, particularly M2-TAMs, tend to exhibit an increased risk of disease recurrence, which is often associated with Th2-type immune response (25, 83).

Hence, the pursuit of anti-tumor strategies targeting TAMs holds the potential to benefit patients across various stages of tumor progression and improve the current treatment failure and high recurrence rates of bladder cancer, which can focus on many targets that can affect the whole life span of TAMs (**Table 1**), and has been validated through many preclinical and clinical research (**Table 2**). These strategies are mainly based on the following mechanisms: (1) TAMs depletion; (2) Reduction of mononuclear/macrophage recruitment; (3) Induction of TAMs to reprogram toward M1-like phenotype; (4) Enhancement of the anti-tumor effects of TAMs.

**Table 1:** TAMs-related therapeutic targets research in bladder cancer.

Therapeutic Targets	Mechanism	Models	Findings	References
**CCL2-CCR2**	The main pathway of TAM recruitment	Bladder cancer cell lines	CCR2 and CCL2 antagonist can inhibit the M-MDSC recruitment and tumor progression	Mu et al. (90) Chen et al. (37) Brana et al. (92)
**CCL2-CCR4**	TAMs recruitment	Mouse model	CCR4+ Tregs were recruited into TME through positive feedback loop with M2-type TAMs by CCL2-CCR4 axis	Chiang et al. (91)
**CXCL12-CXCR4**	TAMs recruitment and polarization	Bladder cancer cell lines	CXCR4 is highly expressed in bladder cancer cells, and interacts with CXCL12 to mediate tumor chemotaxis and invasion	Retz et al. (65)
**GM-CSF**	TAMs recruitment	Mouse model; Clinical research	Administration of GM-CSF can inhibit the growth of bladder cancer	Miyake et al. (95) Burke et al. (97) Packiam et al. (98)
**CSF1/CSF1R**	TAMs recruitment and polarization	Clinical research	CSF1R inhibitor combines with anti-PD-1 therapy leading to the augmented activation of CD8+ T cells and reduction of TAMs	Gomez et al. (100)
**TLR**	TAMs polarization	Mouse model; Clinical research	TLR-3 and TLR-7 agonists can induce polarization to M1-TAMs, and inhibit tumor growth	Smith et al. (110) Camargo et al. (111) Plote et al. (112) Ayari et al. (113)
**HDAC**	TAMs polarization	Bladder cancer cell lines; Mouse model; Clinical research	HDACi alone or combined with anti-PD-1 treatment induced anti-tumor immune response	Burke et al. (116) Grivas et al. (117)
**PI3K**	TAMs polarization	Mouse model; Clinical research	Inhibiting PI3K in FGFR3-mutated tumor achieved promising efficacy by reversing the macrophage phenotype	Ouyang et al. (118)
**CD47-SIRPα**	Inhibits the macrophage-mediated phagocytosis	Mouse model	Blocking SIRPα signaling induced TAMs to transform into anti-tumor properties and attack tumor cells they would otherwise ignore	Kiss et al. (57) Yang et al. (123)

TAM, tumor-associated macrophage; MDSC, marrow-derived suppressor cell; TME, tumor microenvironment; VEGF, vascular endothelial growth factor; GM-CSF, granulocyte macrophage colony-stimulating factor; CSF, colony-stimulating factor; TLR, Toll-like receptor; HDAC, histone deacetylase; PI3K, phosphoinositide 3-kinase; SIRPα, signal regulatory protein α.

**Table 2:** Clinical trials of bladder cancer with agents targeting macrophages.

Targeted Pathways	Agent Names	Combinations	Tumor Types	Clinical Phases	Trial Numbers
Chemotherapy	Zoledronic acid		Bladder cancer	N/A	UMIN000003146
	Docetaxel		Bladder cancer	II	NCT06488222
	Docetaxel	Gemcitabine	Non-muscle invasive bladder cancer	N/A	NCT06374914
	Docetaxel	Epirubicin +/- Gemcitabine +/- Mitomycin	Non-muscle invasive bladder cancer	II	NCT05024734
VEGF	Bevacizumab	Cisplatin +/- Gemcitabine	Bladder cancer	II	NCT00268450
	Bevacizumab	Ipilimumab +/- Pemetrexed +/- TKI +/- Chemotherapy	Locally advanced or metastatic cancers*	III	CTIS2024-513707-14-00
	Bevacizumab		Urothelial carcinoma bladder stage III	III	EUCTR2016-005189-75-CZ
	Bevacizumab	Cisplatin +/- Gemcitabine	Advanced urinary tract cancer*	III	NCT00942331
	Ramucirumab	TRK-950	Advanced solid tumors*	I	NCT03872947
	Ramucirumab	Docetaxel	Bladder, urethra, ureter, or renal pelvis carcinoma	II	NCT01282463
CD40	2141-V11		Non-muscle invasive bladder cancer	I	NCT05126472
	APX005M		Solid Tumors*	I	NCT02482168
	CDX-1140	Pembrolizumab +/- CDX-301 +/- Chemotherapy	Advanced malignancies*	I	NCT03329950
CD47	Evorpacept	Enfortumab vedotin	Urothelial carcinoma	I	NCT05524545
	Magrolimab	Atezolizumab	Urothelial carcinoma	I/II	NCT03869190
TLR-3	Poly ICLC	Nivolumab +/- Synthetic long peptide personalized cancer vaccine	Muscle-invasive bladder cancer	I	NCT06529822
	Poly ICLC	PGV001 +/- Atezolizumab	Bladder cancer	I	NCT03359239
	Poly ICLC	Durvalumab +/- Tremelimumab	Advanced, measurable, biopsy-accessible cancer*	I/II	NCT02643303
TLR-7	Imiquimod		Non-muscle invasive bladder cancer	N/A	ISRCTN65084068
	Imiquimod	TRK-950	Advanced solid tumors*	I	NCT03872947
	Imiquimod		Non-muscle invasive bladder cancer	I	NL-OMON35119
	Imiquimod		Situ bladder cancer	II	NCT01731652
HDAC	Entinostat	Pembrolizumab	Muscle-invasive bladder cancer	II	NCT03978624
	Vorinostat	Docetaxel	Advanced and relapsed solid malignancies*	I	NCT00565227
	Vorinostat		Recurrent or metastatic cancer of the urothelium*	II	NCT00363883
	Mocetinostat		Urothelial carcinoma	II	NCT02236195
PI3K	GSK2636771		Advanced refractory solid tumors, lymphomas, and multiple myeloma*	II	NCT02465060
	Buparlisib		Urothelial carcinoma	II	NCT01551030
	Eganelisib	Nivolumab	Advanced urothelial carcinoma	II	NCT03980041
CSF-1R	Emactuzumab	Atezolizumab	Advanced solid tumors*	I	NCT02323191
GM-CSF	Recombinant fowlpox GM-CSF vaccine		Bladder cancer	I	NCT00072137
	CG0070	Nivolumab	Muscle-invasive bladder cancer	I	NCT04610671
	CG0070		Non-muscle invasive bladder cancer	II	NCT02365818
	CG0070	Pembrolizumab	Non-muscle invasive bladder cancer	II	NCT04387461
	CG0070		Non-muscle invasive bladder cancer	II/III	NCT01438112

*In terms of tumor types, the study specifically included bladder cancer. VEGF, vascular endothelial growth factor; TLR, Toll-like receptor; HDAC, histone deacetylase; PI3K, phosphoinositide 3-kinase; CSF-1R, colony-stimulating factor-1 receptor; GM-CSF, granulocyte macrophage colony-stimulating factor.

### 5.1 TAM depletion strategy

A variety of drugs can be used to eliminate TAM in the tumor microenvironment, such as bisphosphonates (including clodrolip and zoledronic acid) can induce apoptosis of macrophages and reverse their predominant phenotype from M2 to anti-tumor M1 subtype *in vivo (* 84); DNA binding agent Trabectedin induces TAM apoptosis through TNF-associated apoptosis-inducing ligand (TRAIL)/TNF pathway (85, 86). In addition, nanoparticles loaded VEGF siRNA and PIGF siRNA have been demonstrated reducing the quantity of M2-TAMs, thereby inhibiting breast tumor growth and lung metastasis in murine models (87). However, it is worth noting that these drugs have not been investigated in studies pertaining to bladder cancer.

Traditional chemotherapy strategies may contribute to the depletion of TAMs. In a cohort study of patients with MIBC, those treated with gemcitabine/cisplatin showed a notable reduction in M2 macrophages and a significant increase in NK cells (88). Similar finding emerged from a retrospective study of pancreatic cancer, which also found that gemcitabine-treated macrophages had tumor-killing properties, suggesting that chemotherapy may also aid in the reprogramming of TAMs toward an antitumor phenotype (89).

### 5.2 Reduction of mononuclear/macrophage recruitment

In addition to inducing TAM apoptosis, another strategy to reduce macrophages in TME is to reduce the recruitment of mononuclear/macrophage cells. Chemokines associated with TAM recruitment in bladder cancer mainly include VEGF, CCL2, CCL5, CSF and CXCL family molecules. Among them, Bevacizumab (39) and ramucirumab (40), which specifically target VEGF and its receptors, have exhibited promising outcomes in Phase II clinical trials when combined with chemotherapy for locally advanced or metastatic bladder cancer.

The CCL2-CCR2 axis represents the main pathway for TAM recruitment, and further induced the polarization of M1-TAMs to M2-TAMs. In a murine bladder cancer model, the utilization of a selective CCR2 antagonist, RS 504393, effectively blocked TAM recruitment (90). Tregs expressing CCR4 have also been reported to be a receptor for CCL2. Through CCL2-CCR4 axis, CCR4+ Tregs were recruited into TME to induce bladder cancer cell metastasis through positive feedback loop with M2-type TAMs *in vivo*. Utilizing a CCR4 antagonist, C 021 dihydrochloride, can inhibit the activation of CCL2-CCR4 and subsequently reverse the infiltration of CCR4 + Tregs and reduce the incidence of pulmonary metastases (91). Additionally, CCL2 neutralizing antibodies can also reduce tumor cell lymph node metastasis and improve survival in mice by TAM inhibition (37). Furthermore, the CCL2 monoclonal antibody Carlumab has been underwent in Phase I and Phase II clinical trials in solid tumors, demonstrating a highly efficient reduce of macrophages and a significantly delay in tumor regeneration post-chemotherapy (92). Drugs Bindarit and Trabectedin have also been demonstrated to inhibit CCL2 synthesis (6).

Overexpression of CXCL12 prompts monocyte differentiating into immunosuppressive macrophages, correlating with poor clinical outcomes. In a study on mammary adenocarcinoma, tumor-derived CXCL12 was found to be necessary for EGF-induced tumor invasion, and induce the migration of CXCR4-positive macrophages via CXCL12-CXCR4 axis. The efficacy of CXCR4 antagonist AMD3100 has been demonstrated efficacy in reducing the spread and metastasis of breast cancer cells in a murine model (93). Neutralization of CXCL12 using Olaptesed (NOX-A12) also showed promise result in a Phase II trial conducted in multiple myeloma (94). Given the high levels of CXCL12 and CXCR4 observed in bladder cancer tissue, blocking this axis may be a potential therapeutic strategy for bladder cancer. Monoclonal antibodies designed to block CXCR4 have exhibited the capacity to reduce the migration potential of bladder cancer cell lines *in vitro (* 65), although further comprehensive studies are warranted.

GM-CSF is also a factor capable of inhibiting the recruitment of M2 macrophages. Tumor vaccines modified with GM-CSF have been shown to significantly inhibit the growth of MB49 bladder cancer cells in mouse models (95). A conditionally replicating oncolytic adenovirus encoding the cDNA for GM-CSF, CG0070 (96), has been evaluated in several clinical studies for bladder cancer. Intravesical administration of CG0070 demonstrates optimal GM-CSF transgene expression, a favorable safety profile, and promising anti-cancer activity (97, 98).

### 5.3 Induction of TAMs to reprogram toward M1-like phenotype

The substantial presence of TAMs in the tumor microenvironment prompts the need for their reprogramming, shifting them from the tumor-promoting M2 phenotype to the M1 phenotype, thereby stimulating the tumor-killing capability of macrophages and may reverse the immunosuppressive state of TME. Such reprogramming represents a pivotal regulatory mechanism within the context of bladder cancer TME modulation.

Immunotherapeutic approaches have been shown the ability to reprogram M2-type macrophages into M1 phenotype. Clinical investigations in bladder cancer have revealed a significant correlation between the efficacy of immune checkpoint inhibitors, including anti-CTLA-4, PD-1, and PD-L1 antibodies, and the M1/M2-TAM ratio in tumor tissue (99). TAMs regulate tumor cell expression of PD-L1 and CD8+ T cell expression of PD-1, and mediate tumor resistance to PD-1 inhibitors. Moreover, TAMs themselves also expresses PD-1, a molecule that regulates macrophage secretion and inhibits its phagocytosis, and may participate in M2 polarization. These effects can be reversed by PD-1/PD-L1 antibody treatment. Anti-PD-L1 therapy has been demonstrated the capacity to reshape macrophages in a reactive tumor model toward a pro-inflammatory M1 phenotype by elevating IFN-γ levels. Currently, several anti-PD-L1 monoclonal antibodies, including atezolizumab, nivolumab, and pembrolizumab, have been approved by the FDA for the treatment of bladder cancer (13, 100, 101).

Nonetheless, a considerable portion of bladder cancer patients exhibit resistant to anti-PD-1/PD-L1 therapy, for whom combined anti-CD40 therapy proves to be an effective method. In the bladder cancer microenvironment in animal models, CD40 is highly expressed on dendritic cells (DCs) and TAMs, and anti-CD40 agonist antibodies reverse the depletion of CD8+ T cells and activate the tumoricidal activity of TAMs through increasing the secretion of NO and TNF-α (102), thereby yielding significant antitumor effects in mouse models (103, 104). The anti-tumor response of anti-CD40 and anti-PD-1 combination therapy is mainly determined by TAM repolarization and IFN-β secreted by M1-like TAMs. Depletion of TAM in bladder cancer models reduces the efficacy of combination therapy. This combination therapy elicits a robust production of IFN-γ by CD8+ T cells, potentially contributing to the shift in the M2/M1-like phenotype balance of TAMs towards M1-like predominance (105).

The CSF1/CSF1R signaling pathway not only plays an important role in TAMs recruitment, but also induces their polarization toward M2-like phenotypes. Anti-CSF1R monoclonal antibodies (e.g., emactuzumab and RG7155) and small molecule antibodies (e.g., BLZ945 and PLX3397) have been proved to deplete TAMs in tumor models by blocking the CSF1-CSF1R axis and reduce the polarization of TAM into M2-like phenotypes (67, 100, 106). In mouse tumor model, depletion of macrophages with PLX3397, an inhibitor of CSF1R, is capable of enhancing the migration and infiltration of CD8+ T cells into the tumor nests by inhibiting macrophage-mediated T cell exclusion. Although the influence on tumor growth is relatively limited with this single therapy, when combined with anti-PD-1 therapy, it can conspicuously delay tumor progression (52). Similarly, recent clinical trials have shown that monotherapy targeting the CSF1/CSF1R signaling axis exhibit limited efficacy in patients, while its effectiveness is enhanced when combined with anti-PD-1 therapy, leading to the augmented activation of CD8+ tumor infiltration T lymphocytes (TILs) and the reduction of TAMs, thereby resulting in a better objective response rate in bladder cancer patients (100).

TLR, a transmembrane receptor expressed on normal urothelial cells and immune cells, is associated with a positive innate immune response and plays a role in TAM polarization. However, its expression is reduced in tumor cells (107). Binding of TLR to TLR agonists reprogram M2-like macrophages to M1-like phenotypes. As a TLR-2/4/9 agonist, BCG is recognized by the immune cells surrounding the tumor, leading to the activation and nuclear translocation of the transcription factor NF-κB, thus resulting in the secretion of several pro-inflammatory factors such as TNF-α, IFN-γ, IL-6, IL-12, and IL-18. These factors not only promote macrophages infiltration and cytotoxicity (107, 108), and IFN-γ can also induce polarization of macrophages into the proinflammatory M1 subtype (109), which promotes immune rejection of tumor. Similarly, the TLR-3 agonist poly (I:C) and the TLR-7 agonist imiquimod (IMQ, TMX-101) exhibit analogous effects, inducing tumor regression in mouse models of bladder cancer and enhancing the therapeutic response of BCG and PD-1 monoclonal antibody (110–113). These results suggest that TLR agonists could be further investigated as potential treatments of bladder cancer, especially in cases where BCG treatment has failed or enhance its therapeutic efficacy.

Overexpression of histone deacetylase (HDAC) in bladder cancer is associated with macrophage polarization, higher tumor grade, and poor prognosis (16). HDAC inhibitors (HDACi) exert a direct influence on both tumor cells and immune cells by inhibiting the deacetylation of histone and non-histone proteins, and are the first class of epigenetic drugs approved by the FDA for cancer treatment. HDACi TMP195 has demonstrated the ability to reprogram M2-like macrophages into M1-like macrophages, resulting in reduced tumor burden and metastasis (114). Studies have also revealed that HDAC inhibitors such as entinostat (MS-275) and trichostatin-A (TSA) can modulate TME by inhibiting myeloid-derived suppressor cells (MDSCs) and altering the immunosuppressive properties of TAMs, and can enhance the therapeutic efficacy of ICI in the meantime (16, 115). A variety of HDACi have been employed in bladder cancer research. In a mouse model of MB49 bladder cancer, intratumoral or intravenous administration of HDACi CI-994 combined with systemic anti-PD-1 treatment proved effective in inducing a durable anti-tumor immune response, while HDACi SAHA (suberoylanilide hydroxamic acid, vorinostat) showed similar effects in the human SW780 bladder cancer cell line (116). However, a Phase II study involving HDACi Mocetinostat in patients with metastatic bladder cancer yielded limited efficacy and significant toxicity (117).

FGFR3 Alterations are frequently observed in patients with bladder cancer. These mutated FGFR3 receptors possess the capacity to activate the PI3K/Akt signaling pathway in macrophages through the induction of enhanced serine synthesis. This activation subsequently transforms the macrophages into an immunoinert phenotype, thereby creating a cold TME conducive to tumor development. Targeting PI3K in mouse FGFR3-mutated tumor models with the PI3K inhibitor duvelisib achieved promising efficacy by reversing the macrophage phenotype, and its combination with erdafitinib exhibited stronger antitumor activity (118).

Metal immunity may also be involved in the regulation of macrophages in TME. It has been demonstrated that iron overload promotes macrophage polarization towards the M1 phenotype via the ROS/acetyl-p53 pathway, and can simultaneously achieve a multipotent antitumor effect by promoting ferroptosis (119). A series of ION-loaded nanomedicines have been investigated in preclinical research, such as pomegranate-like magnetic nanoparticle (rPAE@SPION) (120) and gel system nanoparticle AuNRs&IONs@Gel (121), that both can induce the polarization of macrophages towards an anti-tumor M1 phenotype in bladder cancer, thereby enhancing the suppression of tumor cells.

Several other drugs have also exhibited the potential to induce TAM repolarization towards M1-like phenotype. Traditional Chinese medicine polyporus has demonstrated notable efficacy with minimal side effects in the treatment of bladder cancer. Research indicated that the water-soluble polysaccharide HPP isolated from polyporus possesses potent immunomodulatory properties. HPP has been shown to induce the transformation of M2 macrophages into M1 subtype *in vitro*, achieved through NF-κB and NLRP3 pathways, and leading to the enhanced secretion of various pro-inflammatory factors by macrophages, thus inhibiting tumor cell growth (9, 12). Furthermore, various drugs, such as docetaxel (3) and CCR5 blocker maravroc (122), have also been elucidated to induce M2-like TAMs repolarization towards M1-like phenotype and inhibit tumor progression.

### 5.4 Enhancement of the anti-tumor effects of TAMs

As previously mentioned, the CD47-SIRPα axis mediated “Don’t eat me” signaling plays a role in tumor evasion from macrophage immune surveillance. Therapies targeting this signaling axis have the potential to fundamentally alter the role of macrophages in tumor biology. Blocking this signaling with CD47 or SIRPα antibodies induced TAMs to transform into an antitumorigenic phenotype, enabling them to attack tumor cells they would otherwise ignore (57, 123). Moreover, the combination of NIR immunotherapy using CD47 antibodies and near-infrared photoimmunotherapy (NIR-PIT) has demonstrated enhanced efficacy in bladder cancer mouse models (56, 124).

In addition, the combination of SHP099, an inhibitor of SHP-2 (the downstream signaling pathway of the CD47-SIRPα axis), along with the CSF1R inhibitor, can lead to the effective repolarization of M2 macrophages towards the M1 phenotype, with superior efficacy compared to monotherapy (125). Another amphipathic supramolecular AK750 targeting SIRPα on macrophages consistently inhibits CSF-1R while blocking the CD47-SIRPα axis, thereby enhancing the repolarization of M2-like TAMs to M1-like phenotype in tumor models, leading to improved anti-tumor and anti-metastasis effects than classical CSF-1R inhibitors (126).

## 6 Conclusion

As the most abundant infiltrating inflammatory cells in the tumor immune microenvironment, TAMs play an important role in the development and metastasis of bladder tumor, and contribute to its resistance and tolerance to anti-cancer drugs. These pro-tumorigenic effects involve a variety of biomolecules and signaling pathways, which may become potential targets for therapeutic intervention of bladder cancer.

A considerable number of drugs targeting TAMs have been validated in preclinical and clinical models across various tumors. Unfortunately, research on targeting TAMs in bladder cancer treatment is still in its early stages. Some drugs, targeting pathways that influence TAMs recruitment and polarization—such as VEGF, TLR, HDAC, and CSF—have already been applied in clinical research for bladder cancer, while many others remain in the preclinical phase. Notably, key pathways with significant effects on TAMs, such as CSF1-CSF1R axis, CCL2-CCR2 axis, and CD47-SIRPα axis, have very few or no clinical studies conducted in the context of bladder cancer. This may offer valuable directions for future research.

Nanomedicine has emerged as a highly promising research field in recent years. Drug-loaded nanoparticle platforms can achieve enhanced targeting specificity, increased infiltration, and extended half-lives. In the context of bladder cancer, several macrophage-targeting nanodrugs are under development (119, 127). However, there remains a scarcity of nanoplatform-based drugs specifically targeting TAMs in bladder cancer, presenting a potential insight for doctors and researchers.
